# Glucose Increase DAGLα Levels in Tanycytes and Its Inhibition Alters Orexigenic and Anorexigenic Neuropeptides Expression in Response to Glucose

**DOI:** 10.3389/fendo.2019.00647

**Published:** 2019-09-20

**Authors:** Alejandra Palma-Chavez, Macarena Konar-Nié, Patricio Órdenes, Felipe Maurelia, Roberto Elizondo-Vega, Karina Oyarce, Sergio López, Joaquin Rojas, Ximena Steinberg, María A. García-Robles, Fernando J. Sepúlveda

**Affiliations:** ^1^Laboratorio de Biología Celular, Departamento de Biología Celular, Universidad de Concepcion, Concepción, Chile; ^2^Laboratorio de Bioquímica y Biología Celular, Departamento de Bioquímica y Biología Molecular, Universidad de Concepción, Concepción, Chile; ^3^Facultad de Medicina y Ciencia, Universidad San Sebastián, Concepción, Chile; ^4^Centro de Estudios Avanzados para la Vida (CREAV), Universidad de Concepción, Concepción, Chile

**Keywords:** feeding behavior, endocannabinoid system, hypothalamus, DAGLα, Tanycytes

## Abstract

The endocannabinoid system (ECS) is composed of a group of Gi-coupled protein receptors and enzymes, producing and degrading the endocannabinoids, 2-arachidonoylglycerol (2-AG) and N-arachidonoyl-ethanolamine (AEA). Endocannabinoid-mediated signaling modulates brain functions, such as pain, mood, memory, and feeding behavior. The activation of the ECS is associated with overeating and obesity; however, the expression of components of this system has been only partially studied in the hypothalamus, a critical region implicated in feeding behavior. Within this brain region, anorexigenic, and orexigenic neurons of the arcuate nucleus (ARC) are in close contact with tanycytes, glial radial-like cells that line the lateral walls and floor of the third ventricle (3V). The specific function of tanycytes and the effects of metabolic signals generated by them on adjacent neurons is starting to be elucidated. We have proposed that the ECS within tanycytes modulates ARC neurons, thus modifying food intake. Here, we evaluated the expression and the loss of function of the 2-AG-producing enzyme, diacylglycerol lipase-alpha (DAGLα). Using Western blot and immunohistochemistry analyses in basal hypothalamus sections of adult rats under several glycemic conditions, we confirm that DAGLα is strongly expressed at the basal hypothalamus in glial and neuronal cells, increasing further in response to greater extracellular glucose levels. Using a DAGLα-inhibiting adenovirus (shRNA), suppression of DAGLα expression in tanycytes altered the usual response to intracerebroventricular glucose in terms of neuropeptides produced by neurons of the ARC. Thus, these results strongly suggest that the tanycytes could generate 2-AG, which modulates the function of anorexigenic and orexigenic neurons.

## Introduction

Food intake and energy expenditure are regulated in part by the hypothalamus (1–8). This brain region has multiple nuclei involved in these functions, in particular, the arcuate nucleus (ARC), which has neuronal populations with antagonistic roles, such as the orexigenic neurons that release neuropeptide Y (NPY) and agouti-related protein (AgRP), and the anorexigenic neurons that release proopiomelanocortin (POMC) (3–8).

Tanycytes are one of the main glial cells present in the basal hypothalamus; they line the lateral lower portion and floor of the third ventricle (3V) with their apical poles facing the ventricular lumen. Tanycytes, through morphological adaptations, can control the secretion of neuropeptides into the portal vessels of the median eminence (ME), regulating the transfer of nutrients (9). They are classified into four main groups based on differences in their localization and gene expression: α1, α2, β1, and β2 (10). α2 and β1-tanycytes are located in the lateral walls of the 3V and contact ARC anorexigenic and orexigenic neurons through their extensive processes. β2-tanycytes cover the floor of the 3V and extend their projections inside the ME. It has been demonstrated that tanycytes sense glucose from the cerebral spinal fluid (CSF) and ME, responding with increased free intracellular calcium (11, 12). Additionally, inhibition of proteins involved in glucosensing in tanycytes, such as glucose transporter 2 (GLUT2), altered neuropeptide expression in response to intracerebroventricular (i.c.v.) glucose injection with consequences in food intake (13–15).

However, the specific function of tanycytes and the effects of metabolic signals generated by them on adjacent neurons remains to be determined. It is possible that tanycytes provide endocannabinoids as an intercellular messenger for modulating neuronal functions by activating type 1 cannabinoid receptors (CB1R), which are widely expressed in the hypothalamus (7). The endocannabinoid system (ECS) is a critical signaling system for food intake and many other homeostatic processes relevant to brain functioning; it consists of endocannabinoid synthesis enzymes (DAGLα and NAPE-PLD), endocannabinoid degradation enzymes, and cannabinoid receptors type 1 and 2 (CB1R and CB2R). The endogenous agonists of CB1R and CB2R, 2-arachidonoylglycerol (2-AG) and N-arachidonoyl-ethanolamine (AEA), are lipids known as endocannabinoids. These compounds show significant affinity for CB1R and CB2R, similar to tetrahydrocannabinol (THC), the main active ingredient present in *Cannabis sativa* (16). For the synthesis of 2-AG, phospholipase Cβ catalyzes the hydrolysis of phosphatidylinositol 4,5-bisphosphate to diacylglycerol, which serves as the substrate for DAGLα and β. Signaling of 2-AG is degraded by monoacylglycerol lipase (MAGL) (17).

It has been demonstrated that CB1R knockout mice exhibit decreased food intake (5) while central administration of cannabinoids promote hyperphagia through activation of CB1R, which could be prevented with the administration of CB1R antagonists (18, 19). Intraperitoneal injection of the CB1R agonist, arachidonyl-2′-chloroethylamide (ACEA), reversed the satiation induced by food ingestion by increasing hunger and inhibiting satiety in mice (20). In rats and humans, administration of THC, a potent CB1 receptor agonist, has been shown to cause hyperphagia and increase preference for pleasant foods (21, 22). Although the ECS in tanycytes has been poorly studied (23), DAGLα immunoreaction has been reported; no other component of the ECS was observed. Furthermore, no further studies have been conducted to date evaluating the expression of the ECS in hypothalamic tanycytes. However, the ECS has been studied in other types of glial cells, such as astrocytes and microglia. In these cell types, the production of 2-AG has a role in neuronal plasticity and inflammatory response (24–27).

In this work, we analyzed the localization of DAGLα in the hypothalamus, showing its localization in α and β tanycytes and ARC neurons. Using both *in vitr*o and *in vivo* approaches, we demonstrated that DAGLα increased its expression in response to increased extracellular glucose levels. Using an adenovirus expressing DAGLα shRNA, we inhibited the expression of this enzyme in tanycytes and demonstrated such inhibition affects the usual response to i.c.v. glucose in terms of orexigenic and anorexigenic neuropeptides produced by neurons of the ARC. We propose that the production of 2-AG by the tanycytes could modulate rat feeding behavior.

## Materials and Methods

### Animals

All animal experiments were performed in accordance with the Guidance on the Operation of the Animals (Scientific Procedures) Act 1986, and all animal studies were approved by the appropriate Ethics and Animal Care and Use Committee of the Universidad de Concepcion, Chile (permit number 2010101A). Adult Sprague-Dawley rats of both sexes weighing 250–300 g and 1-day postnatal were used. Animals were housed in a separate animal room with constant temperature (21 ± 2°C) and a controlled 12-h light/12-h dark cycle; lights were turned on every day at 7:00 a.m. Animals had free access to a standard rodent diet (Lab Diet, 5P00 Prolab RMH 3000, Purina Mills, St. Louis, MO) and tap water.

### Primary Cultures of Rat Hypothalamic Tanycytes

Hypothalamic tanycyte cultures from 1-day postnatal brains were prepared following the method described previously. The hypothalamic region was quickly removed from the brain and further dissected to obtain the tissue containing the ependymal layer. Trypsinized tissue was transferred to tissue culture plates containing minimal essential medium (MEM), (Invitrogen, Carlsbad, CA, USA) with 10% (v/v) fetal bovine serum (FBS) (Thermo Fisher Scientific Inc., Waltham, MA, USA) and 2 mg/mL DNase I (Sigma-Aldrich, St. Louis, MO, USA). Dishes with the highest density of confluent epithelial cells were expanded for subsequent adenoviral transduction to measure cell survival, transduction efficiency, and mRNA and protein expression.

### Culture of HEK 293 Cells

For adenovirus generation, the HEK 293A cell line was used, and HEK293T cells were cultivated in Petri dishes of 10 cm of diameter (Corning® Costar®) with 10 mL of DMEM 25 mM glucose (DMEM-HG) culture medium (Gibco® Invitrogen, Life Technologies) supplemented with 10% v/v FBS (Invitrogen), 0.1 mM non-essential amino acids (Gibco® Invitrogen, Life Technologies), penicillin 100 U/mL, streptomycin 100 μg/mL and fungizone 2.5 μg/mL (Gibco® Invitrogen, Life Technologies). Culture medium was changed every 2 or 3 days.

### RNA Extraction

Total RNA was obtained from primary cultures of tanycytes and hypothalamus of adult rats. RNA was extracted using the guanidine-phenol/chloroform thiocyanate extraction method, homogenizing the samples in 500–1,000 μL of TRIzol® (Invitrogen, Thermo Fisher Scientific Inc.), depending on the number of cells or amount of tissue as instructed by the manufacturer and incubating them for 3 min at room temperature. Then, 200 μL of chloroform was added to the samples, agitated vigorously for 15 s followed by incubation at room temperature for 3 min. Samples were centrifuged at 12,000 g for 15 min at 4°C to separate phases. The aqueous phase containing RNA was recovered, and 500 μL of isopropanol was added; the samples were incubated for 10 min at room temperature and then centrifuged at 12,000 g for 10 min at 4°C. The supernatant was removed, and the RNA pellet was dried for 10 min at room temperature. Finally, the total RNA was suspended in 30 μL water free of RNAse and quantified by measuring absorbance at 260 nm. Extraction purity was evaluated by measuring the ratio at 260/280 nm.

### Reverse Transcription of Total RNA (RT)

cDNA synthesis was performed in a Mastercycler thermal cycler (Eppendorf), using 2 μg of total RNA from each sample. Before the reverse transcription process, 2 μg of total RNA was treated with DNAase (Thermo Fisher Scientific) at 37°C for 30 min to eliminate any possible DNA contamination. For a final volume of 20 μL, the above mixture was incubated with 2.5 mM EDTA for 10 min at 65°C followed by incubation with 0.5 μg Oligo-dT and denatured at 70°C for 5 min. Subsequently, the transcription buffer (50 mM Tris-HCl, pH 8.3, 50 mM KCl, 4 mM MgCl_2_, 10 mM DTT), dNTPs (1 mM each) and 20 U of the RNA inhibitor or Ribolock (Thermo Fisher Scientific) were added, incubating for 5 min at 37°C. Then, 200 U of RevertAid® H Minus M-MuLV (Thermo Fisher Scientific) reverse transcriptase was added to this mixture and incubated for 1 h at 42°C, followed by 70°C for 10 min to stop the reaction. As a negative control, parallel reactions were performed in the absence of oligo-dT and reverse transcriptase enzyme (RT) to detect the presence of contaminating DNA.

### Immunofluorescence

Cells were cultured on 0.2 mg/mL poly-L-lysine-coated glass cover slides (Sigma-Aldrich) in 24-well plates and fixed with 4% paraformaldehyde (PFA) in PBS for 30 min. Immunocytochemistry was performed as previously described (28), using the following primary antibodies: anti-DAGLα (1:200, sc-390409, Santa Cruz Biotech, Santa Cruz, CA) and anti-Vimentin as a tanycyte marker (1:200, AB5733, Millipore, Burlington, MA). Immunohistochemistry was performed using rat brain slices (40 μm) obtained by freezing microtomy after fixation by vascular perfusion with 4% PFA. The slides were visualized using confocal microscopy LSM700 (Zeiss).

### Western Blot

Total protein extracts (50 μg) were separated in denaturing gels and transferred to an immobilon P membrane (Millipore). The membrane was blocked with milk and incubated with the following antibodies for 16 h: anti-DAGLα (1:1,000, sc-390409 Santa Cruz [E-6]), anti-GFP (1: 1,000, sc-8334, Santa Cruz), and anti-β-actin-HRP (1:10,000, sc−47778, Santa Cruz). The membrane was next incubated with the secondary antibody conjugated to HRP (1:5,000, Jackson Immuno-Research) for 2 h. Detection of peroxidase activity was performed by the chemiluminescent detection system in an automated fluorescent/chemiluminescent imaging equipment (Clinx Science Instruments Co., Ltd., Model ChemiScope 3300).

### Real-Time Quantitative Polymerase-Chain-Reaction

Quantification of the cDNA was based on the ΔΔCt method. qRT-PCR was performed using Brilliant II SYBR Green QPCR Master Mix (Agilent Technologies, Wilmington, DE) amplified in the Mx3000P QPCR System thermocycler (Agilent Technologies). Data are presented as relative mRNA levels of the gene of interest normalized to cyclophilin mRNA levels. The following sets of primers were used: cyclophilin, sense 5′-ATA ATG GCA CTG GTG GCA AGT C-3′ and antisense 5′-ATT CCT GGA CCC AAA ACG CTC C-3′; DAGLα, sense 5′-TGA TCT GAC CAT CGC CCT TT-3′ and antisense 5′-AGC GCT GTC TTT CCC TTG TT′; NPY, sense 5′-TGT TTG GGC ATT CTG GCT GAG G-3′ and antisense 5′-CTG GGG GCA TTT TCT GTG CTT TC-3′; and POMC, sense 5′-CTC CTG CTT CAG ACC TCC ATA GAC-3′ and antisense 5′-AAG GGC TGT TCA TCT CCG TTG-3′.

### Glycemia Measurement

Blood samples were collected by puncture in the tail vein of the rat. Glycemia was determined with the Accu-Chek Go glucometer (Roche). We measured the glycemia at the end of the fasting periods and at 1 h after intraperitoneal glucose or saline injection.

### Preparations of Adenoviral shRNA-DAGlα Vectors

The sequence targeting rat DAGLα (GenBank: NN_001005886.1:94-3228) was selected using siDESIGN Center (Dharmacon RNAi technologies), and sequences with homology with other rat coding sequences by BLAST analysis were discarded. The following oligonucleotides were used: sense 5′-TCA ATA AGG TGC TGG AGA ACGC GCC GCA GCT TCT TTC TGT AAC ATT CAA GAG ATG TTA CAG AAA GAA GCT GCT TTT TTT TAA T-3′ and antisense 5′-TTC TCC AGC ACC TTA TTG A-3′. A ring sequence of nine base pairs (TTC AAG AGA) was placed between the sense and antisense strands. Control siRNA oligonucleotides were designed and selected to target β-galactosidase from *E. coli:* sense 5′-CGC GCC AAG GCC AGA CGC GAA TTA TTT CAA GAG AAT AAT TCG CGT CTG GCC TTT TTT TTT TAA T-3′ and antisense 5′-TAA AAA AAA AAG GCC AGA CGC GAA TTA TTC TCT TGA AAT AAT TCG CGT CTG GCC TTG G-3′. Cloning of the expression cassette into the adenoviral shuttle vector was then performed. Briefly, the fragment encoding the H1-promotor, multicloning site (MCS), ubiquitin promoter, EGFP and SV40 polyA from the Fux vector was ligated into the EcoRI and SacI sites of the pDC311 adenoviral shuttle expression vector (Microbix, Ontario, Canada) (14). The shRNA was cloned into the MCS through the AscI and PacI sites. The adenoviral expression system was produced by cotransfecting HEK293A cells with pBHGlox(Δ)E1,3Cre (Admax system, Microbix biosystems Inc. Ontario, Canada) adenoviral genomic DNA and either pDC311-H1-shDAGLα-Ub-EGFP or the pDC311-H1-shbGal-Ub-EGFP expression vectors. The resulting adenoviral expression vectors were titered by EGFP expression using the Adeno-XTM Rapid Titer Kit Protocol (Clontech). After amplification, adenoviral particles were purified using the VirakitAdenoMini-4 kit (Virapur, San Diego, CA), aliquoted and stored at −80°C.

### Adenoviral Transduction *In vitro*

To measure cell survival and transduction efficiency, tanycyte cultures were grown on poly-L-lysine-coated glass cover slides in 24-well plates in MEM supplemented with 10% (v/v) FBS. Cells were transduced with Ad-DAGLαshRNA or Ad-βGalshRNA (control) at 5 × 10^7^ infectious units per mL (IFU/mL). Virus-containing medium was replaced 24 h later with MEM containing 10% (v/v) FBS and incubated for a total of 48, 72, and 96 h. Survival was measured by the trypan blue exclusion assay. Transduction efficiency was calculated as the percentage of total cells obtained using the nuclear marker, TOPRO-3 (1:1,000, Invitrogen) by the number of EGFP-positive cells. Coverslips were visualized by confocal microscopy LSM 700 (Zeiss, Germany) after fixation with PFA.

### Cannula Implantation

Cannulae were stereotaxically implanted into the 3V with the following protocol. Rats were anesthetized with an intraperitoneal injection mix of ketamine-xylazine (90 mg/kg^−10^ mg/kg), and the fur at the top of the head was removed to expose the area to be incised. A hole was drilled in the skull, and a guide cannula (28 gauge stainless steel; Plastics One, Roanoke, VA) was lowered using the following stereotaxic coordinates: anterior-posterior from bregma −3.14 mm, medial-lateral from midsaggital sinus 0.0, and dorsal-ventral from the top of the skull 9.2 mm. The guide cannula was secured to the skull using 3/32 mm mounting screws and dental acrylic. A removable dummy cannula (28 gauge stainless steel; Plastics One, Roanoke, VA) fit into the cannula guide, sealing the opening in the guide cannula throughout the experiments except when it was removed for the injections. Rats were housed individually following surgery and allowed to recover for 5 days before adenovirus administration and starting the experimental procedures.

### i.c.v. Injections of AdshDAGLα and AdshβGal Adenovirus

Rats were anesthetized with isoflurane and then injected into the 3V with 30 μL of 5 × 10^7^ IFU/mL (2.5 μL/min). For neuropeptide expression analysis in response to i.c.v. glucose, cannulated animals were used after 5 days of recovery time. At 72 h post-transduction, the animals were fasted for 48 h until 120 h post-transduction. Subsequently, the animals were anesthetized with isofluorane and injected i.c.v., through the cannula guide, with 20 μL of saline buffer (128 mM NaCl, 3 mM KCl, 1.3 mM CaCl_2_, 1.0 mM MgCl_2_, 1.3 mM NaH_2_PO_4_, 21 mM Na_2_HPO_4_, pH 7,4 and 320 mOsm) or 20 μL of 50 mM glucose prepared in the same buffer [320 mOsm, pH 7,4]. Hypothalamic samples were collected at 2 h post-glucose or saline injection for the mRNA expression analysis.

### Statistical Analyses

For statistical analysis, each treatment was compared to its respective control. Significant differences were determined using the Student's *t*-test and Mann-Whitney *post-hoc U*-test or one-way ANOVA with multiple comparison test. Differences in all experiments were considered significant when *p* < 0.05 using GraphPad Prism 5.0 Software (GraphPad Software Inc., San Diego CA, USA). Results were expressed as mean ± standard error of the mean (SEM), and n refers to the number of animals used.

## Results

### Extracellular Glucose Regulates DAGLα Expression in Tanycytes *In vitro* and *In vivo*

**Figure 1:**
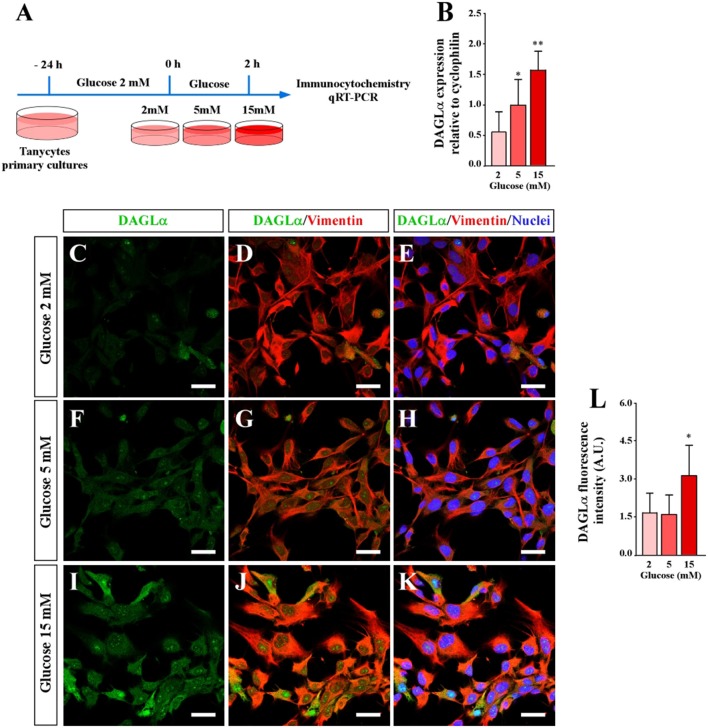
Effect of extracellular glucose on DAGLα expression using primary cultures of tanycytes. **(A)** Scheme of the experimental protocol. **(B)** qRT-PCR analysis DAGLα mRNA expression 2 h post incubation with 2, 5, and 10 mM glucose. **(C–K)** Confocal microscopy of tanycytes incubated in several glucose conditions and stained with anti-DAGLα (green), anti-vimentin (red) and TOPRO-3 as a nuclear marker. **(C–E)** 2 mM glucose. **(F–H)** 5 mM glucose. **(I–K)** 15 mM glucose. Scale bar: 20 μm. **(L)** Quantification of the fluorescent signal associated with DAGLα. Bars represent average ± S.D. *p < 0.05, **p < 0.01.

Immunohistochemical studies report that DAGLα is localized in hypothalamic tanycytes (23). First, we analyzed if primary cultures of tanycytes expressed DAGLα using qRT-PCR and immunocytochemistry analyses at three glucose concentrations (2, 5, and 15 mM) (Figure 1A). These concentrations were selected taking into account that they could be reached in the 3V and increase intracellular calcium in tanycyte cultures (12, 29). qRT PCR data was normalized to cyclophilin and the 5 mM glucose condition. Tanycytes incubated in 2 mM glucose exhibited relative levels of DAGLα expression of 0.53 ± 0.4, while cells incubated in 15 mM glucose exhibited relative levels of 1.28 ± 0.6 (Figures 1B,C,F,I). These data suggest that high extracellular glucose concentrations lead to a significant increase in DAGLα expression. However, vimentin immunoreactivity did not change in response to glucose conditions.

Previous studies have demonstrated that tanycyte cultures exhibited elongated cytoplasmic processes that have a degree of polarity (12, 30) (Figures 1D–L). Immunocytochemical analysis revealed that DAGLα is widely detected in the cytoplasm and nuclei of tanycytes cultured in 15 mM glucose (Figures 1J–L). However, a lower intensity was detected at 2 and 5 mM glucose (Figures 1C–F). The fluorescence associated with DAGLα was quantified and expressed in arbitrary units confirming increased DAGLα expression in tanycytes exposed to 15 mM glucose (Figures 1I–L). These results show that DAGLα expression in tanycytes depends on extracellular glucose concentration, suggesting a role for this enzyme in tanycyte glucosensing.

**Figure 2:**
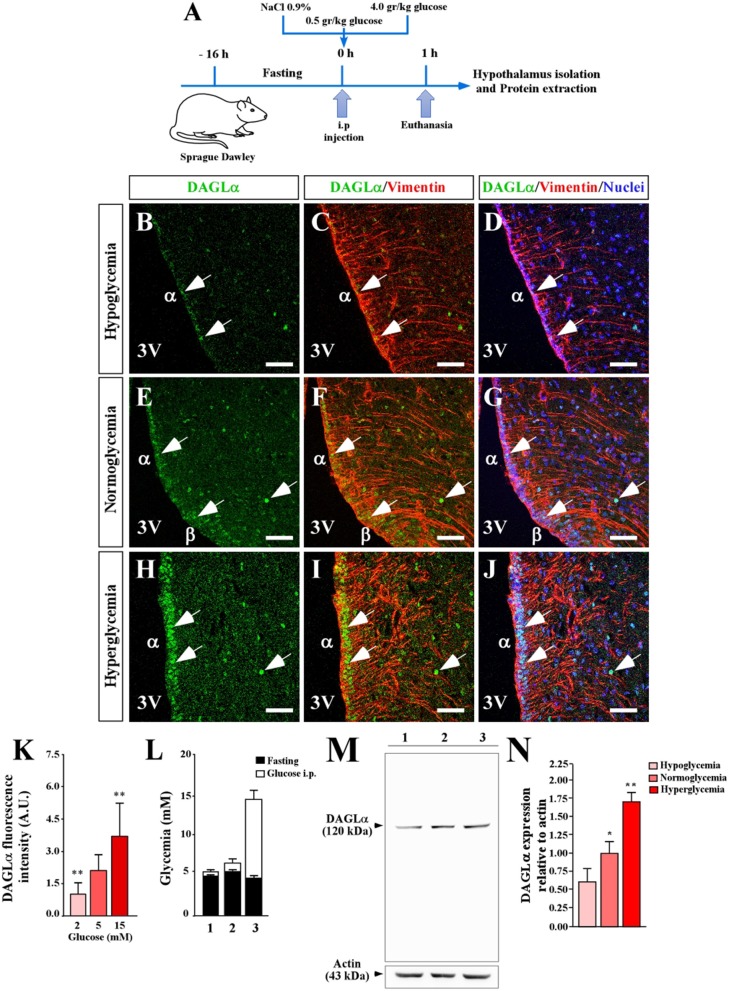
Effect of extracellular glucose on the expression of DAGLα in the basal hypothalamus. **(A)** Scheme of the experimental protocol. **(B–J)** Confocal microscopy of a coronal section (40 μm) of rat hypothalamus stained with anti-DAGLα (green), anti-vimentin (red) and TOPRO-3 as a nuclear marker. Hypoglycemia **(B–D)**, normoglycemia **(E–G)**, and hyperglycemia **(H–J)**. Scale bar: 100 μm. **(K)** Quantitative analysis of nuclear fluorescence for DAGLα in tanycytes expressed in arbitrary units (AU). **(L)** Glycemia recorded after fasting (black bars) and 1 h after glucose injection (white bars). **(M)** Detection of DAGLα in total protein extract (50 μg) of periventricular hypothalamus of adult rats by Western blot analysis, showing the entire gel for demonstrating the specificity of the DAGLα antibody. **(N)** Densitometric analysis of the Western blot for DAGLα. In L and M 1, 2 and 3 are hypoglycemia, normoglycemia, and hyperglycemia conditions, respectively. Bars represent average ±S.D. 3V: third ventricle. *p < 0.05, **p < 0.01. Bar size 80 μm.

To know if the levels of DAGLα in the hypothalamic region are dependent on the glycemic condition, we modified the glycemia of adult animals using a protocol previously reported and briefly described in Figure 2A (29), in which glucose concentration in the 3V reaches nearly 10 mM in the hyperglycemic condition. Fasting, normoglycemic and hyperglycemic conditions were induced by 16 h fasting, 1 h post-intraperitoneal injection of saline, or 0.5g/kg or 4g/kg body weight glucose, respectively (Figure 2L). In hypoglycemia (4.5 ± 0.5 mM), a slight reaction by DAGLα was detected and mainly localized in the ventricular area (tanycytes) and only in very few parenchymal cells of the ARC (Figures 2B–D). In normoglycemia (7.0 ± 1.1 mM), a slight but greater intensity of immunoreaction than observed in hypoglycemia was detected, mainly in α- and β-tanycytes (Figures 2E–G); in some cases, evident nuclear localization was detected as shown by co-labeling with the nuclear marker. In hyperglycemia (14.5 ± 1.1 mM), an intense immunoreaction was detected although the intensity in α-tanycytes was less than that detected in β-tanycytes and parenchymal cells (Figures 2H,I) with evident nuclear localization as shown by co-labeling with the nuclear marker (Figure 2J). The intensity of ventricular fluorescence associated with DAGLα was quantified, showing significant changes compared to the normoglycemic condition (Figure 2K). Finally, using total protein lysates from microdissected basal hypothalamus as well as the cerebellum, we demonstrated that the DAGLα antibody recognized a unique band of 120 kDa (Figure 2M), which has been previously reported (31). Importantly, our methodology to increase plasma glucose levels produced a significant increase in total DAGLα levels (Figures 2M,N). Actin was used as a loading control and for normalizing the samples for quantification. Taken together, these results indicate that glucose increases DAGLα in tanycytes. However, it cannot be ruled out that there is also an increase in the parenchyma although the immunohistochemistry data suggest that this increase is lower than in the ventricular region.

### Development of Adenoviral Particles to Modulate DAGLα Expression of Tanycytes *In vivo*

**Figure 3:**
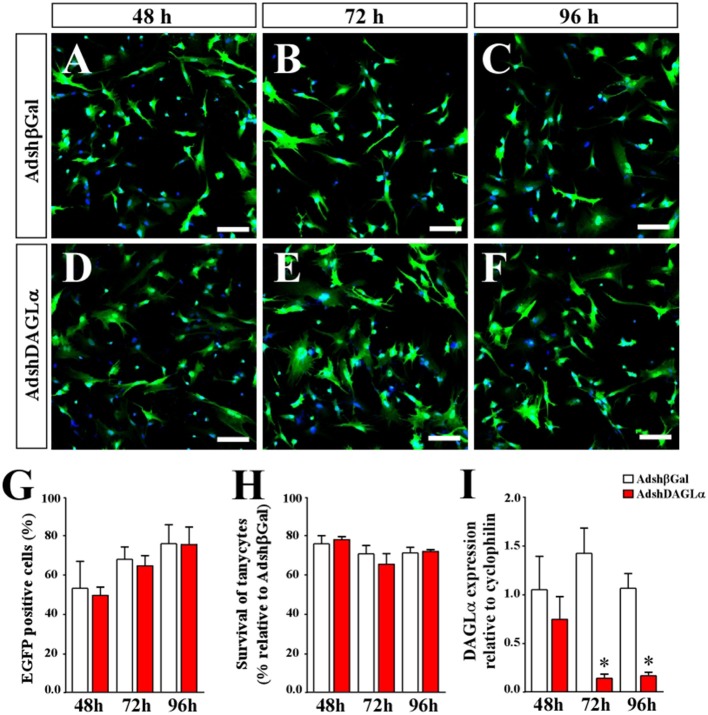
Evaluation of adenoviral particles carrying DAGLα-specific shRNAi. **(A–F)** Representative images for *in vitro* cultures of tanycytes infected with AdshDAGα and Adshβgal as a control adenovirus for 48–96 h. **(G)** Quantification of transduction percentages by the adenoviral particles used in **(A–F)**. **(H)** Quantification of cell survival shown as the percentage of transduced tanycytes with AdshDAGα and Adshβgal at 48–96 h post-transduction. **(I)** mRNA levels for DALGα in transduced tanycytes with AdshDAGα and Adshβgal at 48–96 h post-transduction. *N* = 4 independent experiments for viability and transduction. Bars represent average ± S.D. Bar size = 20 μm. *p < 0.01.

After adenoviral production, *in vitro* tests were performed to evaluate the transduction capacity, cytotoxic effect and ability to decrease mRNA levels of DAGLα. The adenoviral particles, AdshDAGLα or Adshβgal, transduced nearly 80% of the cells at 96 h of infection (Figures 3A–G) without presenting significant toxicity between 48-96 h (Figure 3H). Furthermore, AdshDAGLα significantly decreased the mRNA levels of DAGLα in tanycyte cultures at 72 h post-transduction (Figure 3I).

The adenoviral serotype corresponded to number 5, and has been described by our laboratory as well as others for presenting positive tropism for ependymal cells and not astrocytes and neurons (13–15, 32–36).

**Figure 4:**
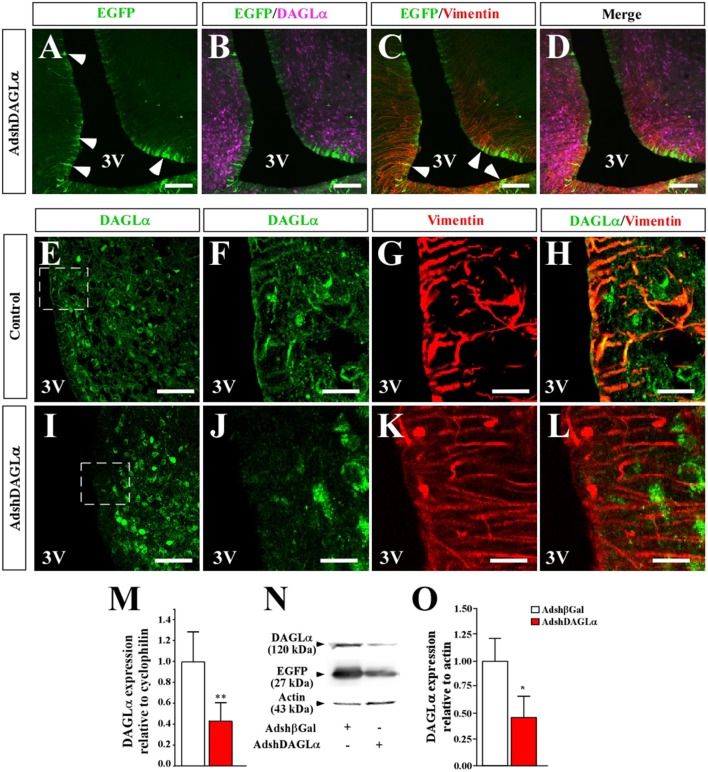
Effect of i.c.v-injected AdshDAGLα in the 3V on the expression of hypothalamic DAGLα. **(A–D)** Frontal sections of the hypothalamus (40 μm) in which the EGFP fluorescence (**A–D**, green), immunoreactivity for DAGLα (**B,D**, purple) and vimentin (**C,D**, red) are shown in cells transduced with AdshDAGLα. **(E–H)** High magnification images showing immunoreactivity for DAGLα (**E,F,H**, green) and vimentin (**G,H**, red) in control animals. **(I–L)** High magnification images showing immunoreactivity for DAGLa (**I,J,L**, green) and vimentin (**K,L**, red) in AdshDAGL-transduced rats. Inset in **(I)** compared with inset in **(E)** show the decrease in reactivity for DAGLα in the ventricular region and not in the parenchymal region. **(M)** mRNA quantification for DAGLα by qRT-PCR (*n* = 6). **(N)** Representative Western blot for all conditions evaluated in **(B)** (*n* = 4). **(O)** Western blot quantification. Bars represent average ± S.D. Bar size **(A–D)** 200 μM, **(E–L)** 50μm. *p < 0.05, **p < 0.01.

We next evaluated the ability of AdshDAGLα or Adshβgal to decrease DAGLα levels in tanycytes *in vivo*. Following injection of AdshDAGLα or Adshβgal (30 μL) directly into the 3V of adult female rats, we evaluated DAGLα expression by qRT-PCR and Western blot analyses after 120 h. Animals injected with AdshDAGLα into the 3V had 60% lower DAGLα mRNA levels than those injected with Adshβgal (Figures 4M,N). In turn, the total protein levels of DAGLα in the basal hypothalamus were 55% lower in animals injected with AdshDAGLα compared to those injected with Adshβgal at 120 h, indicating that adenoviral infection significantly decreased *in vivo* DAGLα levels in tanycytes (Figures 4C,D).

As DAGLα is localized in tanycytes and parenchymal cells (possibly neurons), it was important to evaluate which cell types were transduced with AdshDAGLα and have reduced DAGLα expression. Following injection of adenoviral particles carrying DAGLα shRNA and an *EGFP* reporter gene into the 3V of female rats, frontal sections of the basal hypothalamus from transduced animals were analyzed by immunofluorescence and confocal microscopy to detect EGFP (green) and anti-DAGLα (magenta) and anti-vimentin (red) immunoreactivity (Figures 4A–D). EGFP expression was detected in ventricular cells with elongated processes, which due to its location and co-labeling with vimentin corresponded to tanycytes (Figures 4A,C). EGFP fluorescence was not detected in ARC parenchymal cells (Figure 4A). Localization of DAGLα was evaluated in hypothalamic slices of control and transduced animals (Figures 4E–L). In frontal sections of control animals, higher magnification images were used to demonstrated that DAGLα-positive cells (green) were also positive for vimentin (Figures 4G,H). However, in rats transduced with AdshDAGLα (Figures 4K,L), a very low reaction associated with DAGLα was detected in the ventricular region, and no co-labeling with vimentin was observed. Moreover, in the parenchyma, DAGLα immunoreaction was detected with the same intensity as that observed in control rats. DAGLα mRNA and protein levels were significantly decreased (Figures 4M,O). These results indicate that AdshDAGLα selectively inhibits DAGLα expression in tanycytes.

### Altered Hypothalamic Neuropeptide Production in Animals Injected With AdshDAGLα Into the 3V

**Figure 5:**
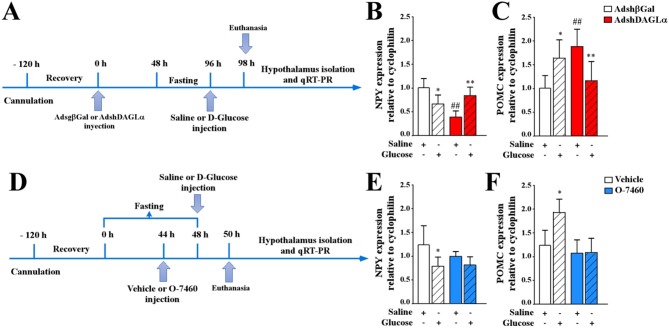
Effect of AdshDAGLα injected in the 3V on the expression of hypothalamic neuropeptides critical for food intake. **(A)** Experimental protocol using adenoviruses. Adult female rats were stereotaxically cannulated into the 3V. After 120 h of recovery, the rats were injected with AdshβGal or AdshDAGL. At 48 h post-injection, the rats were fasted for 48 h and subsequently injected with saline buffer or 50 mM D-glucose (dashed bars). At 2 h post-3V i.c.v. Injection, the animals were sacrificed, and the hypothalamus was dissected for RNA extraction and qRT-PCR analysis. **(B,C)** qRT-PCR analysis of NPY **(B)** and POMC **(C)** neuropeptide expression in rats transduced with AdshβGal (open bars) or AdshDAGLα (red bars). **(D)** Experimental protocol using the O-7460 inhibitor. At 48 h post-injection, the rats were fasted for 44 h and injected with vehicle (open bars) or inhibitor (blue bars). After 2 h, they were injected with saline buffer or 50 mM D-glucose (dashed bars). At 2 h post-3V i.c.v. injection, the animals were sacrificed, and the hypothalamus was dissected for RNA extraction and qRT-PCR analysis. **(E,F)** qRT-PCR analysis of NPY **(E)** and POMC **(F)** neuropeptide expression. ANOVA ^*,#^*p* < 0.05; ^**,##^*p* < 0.01. Bars represent average ± S.D for 5–6 independent experiments.

Food intake is in part regulated by the release of neuropeptides produced in the ARC, namely the orexigenic neuropeptide, NPY, and the anorexigenic neuropeptide, POMC. Therefore, we next determined whether the 2-AG-producing enzyme in tanycytes potentially had any effect on the expression levels of these neuropeptides in response to increased glycorrhachia. It has previously been reported that injection of 50 mM glucose into the 3V generates a neuronal response mediated by changes in anorexigenic and orexigenic neuropeptide mRNA expression (13–15, 37). Under normal conditions, glycorrhachia decreases the expression of NPY and increases the expression of POMC neuropeptides. As shown in the experimental scheme shown in Figure 5A, we examined if this glucose response was maintained in DAGLα-inhibited rats. Neuropeptide expression was measured by qRT-PCR in Adshβgal (white bars) and AdshDAGLα (red bars) knockdown animals after 2 h of saline (open bars) or D-glucose (dashed bars). After glucose injection, the Adshβgal group had reduced NPY expression (45% reduction) compared to animals injected with saline buffer (Figure 5B, open-dashed bar). In contrast, POMC expression was increased by 60% compared to animals injected with saline buffer (Figure 5C, open-dashed white bar), which was similar to that previously reported with this experimental approach (13–15).

In contrast, DAGLα-inhibited rats showed an erratic response; in fasting conditions, reduced NPY expression was observed (Figure 5B, red-open bar), increasing in response to glucose (Figure 5B, red open-dashed bar) compared to those transduced with the control virus. These results indicate that glial DAGLα inhibition decreased NPY expression in fasting conditions, but not in response to i.c.v. glucose. In AdshDAGLα-transduced rats, POMC expression in the fasting condition was approximately 50% greater compared with that observed with the control adenovirus (Figure 5C, white and red open bars). However, the response to glucose differed from that observed in the control group with POMC decreasing by 40% in AdshDAGLα-transduced rats while increasing in control conditions (Figure 5C, red-dashed bar). These results suggest that inhibition of 2-AG production by tanycytes may increase hunger and decrease satiety. Because DAGα is not only expressed by tanycytes but also by parenchymal cells, we decided to test the effect of the pharmacological inhibitor, O7460, which inhibits neuronal and tanycyte DAGLα when injected into 3V due to its hydrophobic nature using the protocol shown in Figure 5D. Whereas the control animals (without inhibitor) showed the expected responses (Figures 5E,F, white bars), neither NPY nor POMC levels in O7460-treated animals in fasting differed with the control; however, there was a total loss in the response to increased glycorrhachia.

## Discussion

Different mechanisms involving the participation of the central nervous system (CNS) have been described in the regulation of food intake. However, much remains to be known about the role of the ECS in this function, mainly if tanycytes can produce endocannabinoids that may, in turn, impact feeding behavior through hypothalamic neurons.

Despite the great amount of evidence describing the role of CB1R in modulating food intake (5, 18–22), very little is known about the participation of the enzymes involved in endocannabinoid synthesis, such as DAGLα in these processes. To the best of our knowledge, only one article has been published showing DAGLα expression in hypothalamic tanycytes (23). To date, it is accepted that CB1R activation in the CNS is associated with an increased food intake (4–6). ARC POMC and NPY neurons are innervated by CB1-expressing GABAergic terminals. In the hypothalamus, high levels of immunoreactivity for CB1R have been found in hypothalamic nuclei in many areas, including the ARC, paraventricular nucleus (PVN), ventromedial nucleus (VMN), dorsomedial nucleus (DMN) and in the lateral hypothalamic area (LHA) (8). Interestingly, POMC neurons in the ARC express CB1R both in the soma (6) and in GABAergic and glutamatergic presynaptic terminals (5). In contrast, CB1R expression in the soma of orexigenic neurons producing NPY/AgRP has not been detected, but using electron microscopy and immunolabeling, it was detected in GABAergic terminals (7).

Using *in vitro* and *in vivo* strategies, we demonstrated increased DAGLα expression in response to increases in extracellular glucose levels, a key molecule in energy metabolism. It has been demonstrated that the glycemic condition that increased DAGLα levels *in vivo* also increased it in the 3V such that it was higher than that of the lateral ventricle, which could be attributed that the β2-tanycytes are in contact with the ME where capillaries are fenestrated. Furthermore, this area has been shown to alter its permeability in response to dietary changes (9). Finally, we suggest that DAGLα expressed by tanycytes could modulate neurons of the ARC to regulate feeding behavior.

Glucose is a fundamental molecule for energy metabolism and is capable of altering food intake in animals (13–15, 37). Components related to glucose metabolism, such as glucosensing enzymes, glucose transporters (GLUTs) and response elements of these proteins have been described to be present in the ARC. For example, it has been described that glucose transporter 2 (GLUT2), monocarboxylate transporters 1 and 4 (MCT1 and 4) and enzymes, such as glucokinase (GK) and GK-regulatory protein (GKRP), are expressed in hypothalamic tanycytes and play a relevant role in food intake (14, 15, 28–30, 38). In this sense, our study demonstrates that increased extracellular glucose induces DAGLα expression in tanycytes, which has a consequent increase in the production of 2-AG, producing an effect in hypothalamic neurons expressing CB1R, the main pharmacological target of 2-AG in this brain area.

Despite finding that extracellular glucose-induced an increase in the expression of the endocannabinoid-producing enzyme, DAGLα, it was not possible to evaluate the direct production of 2-AG under different glucose concentrations either *in vitro* or *in vivo*. However, RNAseq experiments from *in vitro* tanycyte cultures showed that this glial cell presents all the components necessary to produce and degrade 2-AG (European nucleotide archive (ENA), access number PRJEB28405). For example, it has an important number of DAGLα copies, the MAGL enzyme, purinergic receptor 2 (P2Y), and a GPCR that increases the levels of PLC-β it also raises the levels of intracellular Ca^2+^. Previous studies from our laboratory as well as others have shown that an increase in extracellular glucose in tanycyte cultures of 10 mM produces an increase in Ca^2+^, a signal required for 2-AG synthesis (12, 27). Here, we demonstrated that DAGLα expression is increased in response to high glucose levels *in vitro* and *in vivo*. It is possible that both mechanisms can increase 2-AG production in tanycytes, which could inhibit POMC neurons and produce hyperphagia.

Our data show that the adenovirus used in our study (serotype 5) together with the site of injection (3V) generate positive tropism for ependymal cells (i.e., tanycytes), and neurons and astrocytes were not transduced, which is in agreement with previous studies described by our group and others (13–15, 32–36, 39).

Fluctuation in endocannabinoid levels between male and female rats have been reported in several brain areas. Specifically, Bradshaw et al. found no significant differences in anandamide levels between male and female rats; however, 2-AG was higher in the female pituitary gland and interestingly in the hypothalamus (40). Because we performed our study with female rats, it may have contributed to obtaining more evident results. Thus, further studies are necessary to evaluate if male rats show a similar response.

The animals injected with AdshDAGLα exhibited an altered response to fasting since NPY levels were lower, and POMC mRNA expression was higher than the animals injected with the control adenovirus. These results suggest that DAGLα inhibition could reduce the production of 2-AG in tanycytes and, therefore, block the usual response of these neurons to lengthy fasting conditions (33, 37). Similarly, the response to glucose in DAGLα-inhibited animals produced an opposite effect compared to animals transduced with Adshβgal (i.e., increased NPY and decreased POMC mRNA expression). Both results suggest that the inhibition of DAGLα could contribute to reduced hunger. Intriguingly, POMC levels in DAGLα-inhibited animals injected with glucose were smaller than those observed in fasting. Although the implications of these last data are not clear, it is consistent with our results indicating that glucose increases DAGLα expression. Finally, the use of a neuronal and tanycyte DAGLα inhibitor (O7460) in the 3V altered the production of hypothalamic neuropeptides. Given its hydrophobic nature, it is possible that the inhibitor targeted both ventricular and parenchymal cells that express DAGLα, producing a total loss in glucose response.

Taken together, our data suggest that the production of 2-AG is carried out in different cell types of the basal hypothalamus including tanycytes, which modulate the activity of the neuroendocrine neurons of the ARC. Tanycytic DAGLα and its ligand, 2-AG, may increase food intake, which is in agreement with the hyperphagia detected in marijuana smokers. The increased DAGLα expression induced by high extracellular glucose could also reflect a concentration-dependent effect of 2-AG over anorexigenic or orexigenic neurons, which need to be confirmed through further investigations.

## Data Availability Statement

The datasets for this manuscript are not publicly available because We do not have it deposited in any database, but we can send it to all researchers who request it. Requests to access the datasets should be directed to fersepul@udec.cl.

## Ethics Statement

Bioethics Committee, Faculty of Biological Sciences, Universidad de Concepción, Chile.

## Author Contributions

AP-C, MK-N, and FS conceived the experiments and designed the experiments. AP-C, MK-N, FM, and PÓ performed the experiments. AP-C, MK-N, XS, SL, RE-V, and KO analyzed the data. MG-R, JR, and FS contributed to reagents, materials, and analysis tools. FS, RE-V, KO, and MG-R wrote the article. RE-V, KO, and MG-R critically revised the manuscript. All authors have approved the final version of the manuscript and agree to be accountable for all aspects of the work in ensuring that questions related to the accuracy or integrity of any part of the work are appropriately investigated and resolved. All persons designated as authors qualify for authorship, and all those who qualify for authorship are listed.

### Conflict of Interest

The authors declare that the research was conducted in the absence of any commercial or financial relationships that could be construed as a potential conflict of interest.
